# Proteasomal inhibition potentiates drugs targeting DNA topoisomerase II

**DOI:** 10.1016/j.bcp.2015.12.015

**Published:** 2016-03-01

**Authors:** Ka C. Lee, Rebecca L. Bramley, Ian G. Cowell, Graham H. Jackson, Caroline A. Austin

**Affiliations:** a Institute for Cell and Molecular Biosciences, Newcastle University, Newcastle upon Tyne NE2 4HH, United Kingdom; b Institute for Cellular Medicine, Newcastle University, Newcastle upon Tyne NE2 4HH, United Kingdom

**Keywords:** DNA topoisomerase II, Proteasome, Mitoxantrone, Velcade, MG132

## Abstract

The reaction mechanism of DNA topoisomerase II (TOP2) involves a covalent double-strand break intermediate in which the enzyme is coupled to DNA via a 5′-phosphotyrosyl bond. This normally transient enzyme-bridged break is stabilised by drugs such as mitoxantrone, mAMSA, etoposide, doxorubicin, epirubicin and idarubicin, which are referred to as TOP2 poisons. Removal of topoisomerase II by the proteasome is involved in the repair of these lesions. In K562 cells, inhibiting the proteasome with MG132 significantly potentiated the growth inhibition by these six drugs that target topoisomerase II, and the highest level of potentiation was observed with mitoxantrone. Mitoxantrone also showed the greatest potentiation by MG132 in three Nalm 6 cell lines with differing levels of TOP2A or TOP2B. Mitoxantrone was also potentiated by the clinically used proteasome inhibitor PS341 (Velcade). We have also shown that proteasome inhibition with MG132 in K562 cells reduces the rate of removal of mitoxantrone or etoposide stabilised topoisomerase complexes from DNA, suggesting a possible mechanism for the potentiation of topoisomerase II drugs by proteasomal inhibition.

## Graphical abstract

**Figure:**
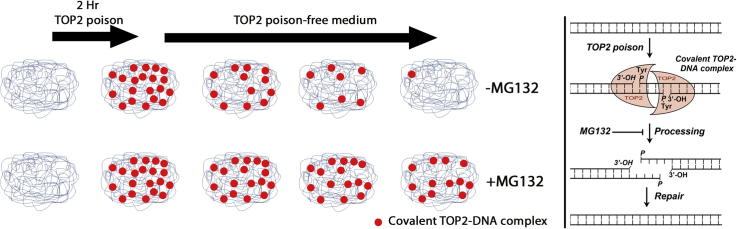

## 1 Introduction

Type II DNA topoisomerases (TOP2s) play a role in several cellular processes including replication, transcription, chromosome condensation and segregation and permit the alteration of DNA topology by allowing one double-stranded DNA segment to pass through another. They achieve this by introducing an enzyme-bridged double-strand break into the first DNA segment, where each monomer of the dimeric enzyme remains covalently attached to the ends of the DSB via a 5′-phosphotyrosyl linkage. The second DNA segment is then “passed” through the enzyme-bridged DNA gate, and the break is re-ligated. The enzyme-bridged gate is normally a short lived intermediate, but a group of drugs, known as “TOP2 poisons” inhibit the religation step resulting in the formation of an unusual type of DSB in which the TOP2 protein remains covalently linked to the DNA. These breaks are cytotoxic, hence the utility of TOP2 poisons such as etoposide, epirubicin and mitoxantrone in cancer therapy. Cells deficient in KU or LIG4 are extremely sensitive to TOP2 poisons, as are cells treated with the DNA-PK_cs_ inhibitor NU7026, implicating NHEJ in the repair of TOP2-induced DSBs [1], [2], [3], [4], [5], [6]. However, TOP2-linked DSBs do not activate DNA-PK in vitro [7] and various lines of evidence suggest cellular processing is required before TOP2-induced breaks elicit a DNA damage response [8], [9]. A number of potential mechanisms exist to remove TOP2 adducts from DNA to allow repair by NHEJ, including cleavage by an AP lyase activity such as KU [2], [10]; removal of the DNA end bearing the TOP2 by a nuclease such as MRE11 [11], [12], [13] or proteolysis of the TOP2 protein followed by the action of a 5′-tyrosyl DNA phosphodiesterase, TTRAP/TDP2 [14], [15], [16], [17].

A number of studies have implicated the proteasome pathway in regulating TOP2 protein levels, although the precise situation is complex and probably depends on cell type and the nature of the stresses that cells are exposed to in different experimental situations. Two topoisomerase orthologues exist in mammalian cells, *TOP2A* and *TOP2B* [18], [19], [20] and both are targeted by topoisomerase poisons such as etoposide [21]. TOP2A but not TOP2B is degraded in a cell cycle dependent manner in a way that appears to involve the ubiquitin–proteasome pathway [22] and TOP2A degradation has also been observed in a number of conditions that generate cellular stress, including adenovirus infection, glucose starvation and oxidative stress [23], [24], [25], [26], [27]. The degradation of TOP2A is ubiquitin and ATP dependent, and a number of ubiquitin ligases including BRCA1, FBW7, MDM2, BMI1/RING1A and ECV have been reported to associate with TOP2A [26], [27], [28], [29], [30], [31]. TOP2 poisons trigger proteasome dependent decreases in TOP2 protein levels. It has been reported that etoposide and teniposide trigger reduction of TOP2A and TOP2B protein [9], [28], [30], [32], [33], whilst ICRF-193 (a catalytic inhibitor) triggers proteasome dependent degradation of only TOP2B [34], [35]. Stabilisation of TOP2 by proteasomal inhibition has been reported to overcome resistance to TOP2 poisons [36], [37]. Here we show that inhibition of the proteasome with MG132 or PS341 potentiates the growth inhibition by some drugs targeting topoisomerase II. In addition, we show that in K562 cells proteasomal inhibition by MG132 reduces the rate of removal of etoposide or mitoxantrone stabilised covalent topoisomerase II complexes from the genomic DNA following drug removal from the media.

## 2 Material and methods

### 2.1 Cell lines

The CML cell line K562, the human pre-B cell line Nalm-6 and the *TOP2A^+/−^* and *TOP2B^−/−^* derivatives of Nalm-6 [38] were cultured in RPMI 1640 medium (Life Technologies, UK) containing 10% foetal bovine serum (Life Technologies, UK) and antibiotics (Life Technologies, UK). Cells were maintained at 37 °C in a humidified atmosphere containing 5% CO_2_. Nalm-6 and its derivatives have a normal p53 status [39], whilst K562 is null for p53 [40], [41].

### 2.2 Growth inhibition assays

Cells were seeded in 96 well plates (Greiner-Bio-ONE, UK) and incubated at 37 °C, 5% CO_2_ for 24 h prior to drug treatment (2000 cells per well for K562 or 10,000 cells per well for Nalm 6 cell lines). Cells were then treated with varying concentrations of anti-topoisomerase II drug alone (Sigma Aldrich, UK) or in combination with the proteasome inhibitor MG132 (Sigma) or PS341 (Cambridge Bioscience, UK) and incubated for 120 h. 50 μL XTT reagent (50:1 XTT reagent to electron coupling reagent, XTT Cell Proliferation kit, Roche, UK) was added per well and cells were incubated for a further 4 h. Absorbance values were obtained using the Bio-Rad 550 Microplate Reader (Bio-Rad, USA) and analysed using GraphPad Prism software (GraphPad Software, USA), version 4.03. Growth inhibition values were determined by setting the values obtained with no drug as 100% for the etoposide-alone data and with MG132/PS341 alone as 100% for the etoposide plus MG132/PS341 data.

The IC_50_ values (concentration at 50% growth inhibition) of anti-topoisomerase II drug alone versus IC_50_ of drug in combination with proteasome inhibitor were used to calculate potentiation factors (Pf_50_). The inhibitory concentration of TOP2 poison in the presence of proteasome inhibitor was divided by the concentration of TOP2 poison alone for each separate experiment. The mean Pf_50_ values in the tables represent the mean of at least 3 individual Pf_50_ values.

### 2.3 In vitro trapped in agarose DNA immunostaining (TARDIS)

TOP2 adducts on genomic DNA were generated by treating K562 cells with 100 μM etoposide or 5 μM mitoxantrone for 2 h prior to embedding cells in agarose on microscope slides (Lonza, USA). To inhibit the proteasome, cells were treated with 50 μM MG132. Cells were collected at the times shown after drug removal and TOP2A and TOP2B complexes were quantified by TARDIS analysis as previously described [42], [43], [44]. Briefly, cells were mixed with molten LMP agarose (Lonza, USA) and spread thinly on slides. Agarose embedded cells were then extracted with 0.1% SDS and 1 M NaCl leaving “nuclear ghosts” consisting of genomic DNA coupled to any TOP2 protein-DNA complexes. TOP2 complexes were then detected by quantitative immunofluorescence from several fields of cells per slide. Microscopy was carried out using an Olympus IX81 motorised microscope fitted with an Orca-AG camera (Hamamatsu) and suitable narrow-band filter sets. Images were analysed using Volocity software (Perkin-Elmer). Experiments were carried out at least in triplicate and data are presented as mean of means obtained for each replicate for each treatment ± SEM. For the data in Fig.9A, rabbit polyclonal antibodies 18511α and 18513β were employed [45], and for the data in Figs. 9B and 10, antibodies 4566-TOP2A and 4555-TOP2B were used. 18511α was raised in-house to recombinant human TOP2A generated in yeast, whilst 18513β, 4566-TOP2A and 4555-TOP2B were raised to GST-TOP2 C-terminal domain fusion proteins generated in bacteria.

### 2.4 Standard immunofluorescence

Cells were plated in PBS (Life Technologies, UK) onto poly lysine-coated slides (VWR, UK) and after allowing 10 min for cells to adhere, they were fixed in PBS containing 4% paraformaldehyde (Sigma, UK). Immunofluorescence was carried out using rabbit anti-TOP2A (4566) and mouse anti-TOP2B (MAB6348, R&D Systems) and Alexa-488 and Alexa-594 coupled anti-rabbit and anti-mouse secondary antibodies respectively (Life Technologies, UK) as described [46].

### 2.5 Western blotting

Cells were washed in ice cold PBS and pelleted in 2 ml microfuge tubes. Whole-cell extracts were prepared and Western blotting was performed as described previously [47]. Poly K48-linked ubiquitin was detected with the rabbit monoclonal APU2 (Merck-Millipore, UK) [48]. Autorads were quantified using a GelDoc EX imager (Bio-Rad). Blots were stripped and re-probed for actin, and APU2 signals were normalised to actin.

## 3 Results

### 3.1 The proteasome inhibitor MG132 potentiates the growth inhibitory effects of anti-topoisomerase II drugs in K562 cells

**Fig. 1:**
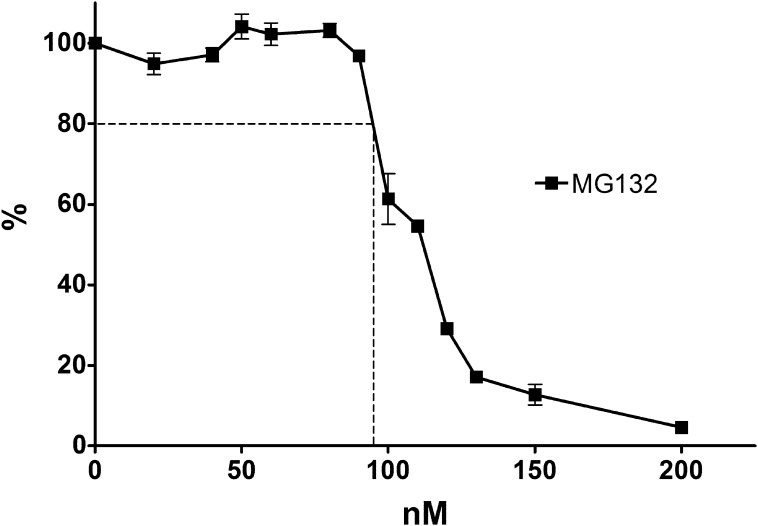
MG132 dose response in K562 cells. The IC_20_ of MG132 in K562 cells was determined by growth inhibition assays. Cells were treated with increasing concentrations of MG132 and stained with XTT after 5 days of incubation. Error bars represent the mean ± SEM of 4 separate experiments.

**Fig. 2:**
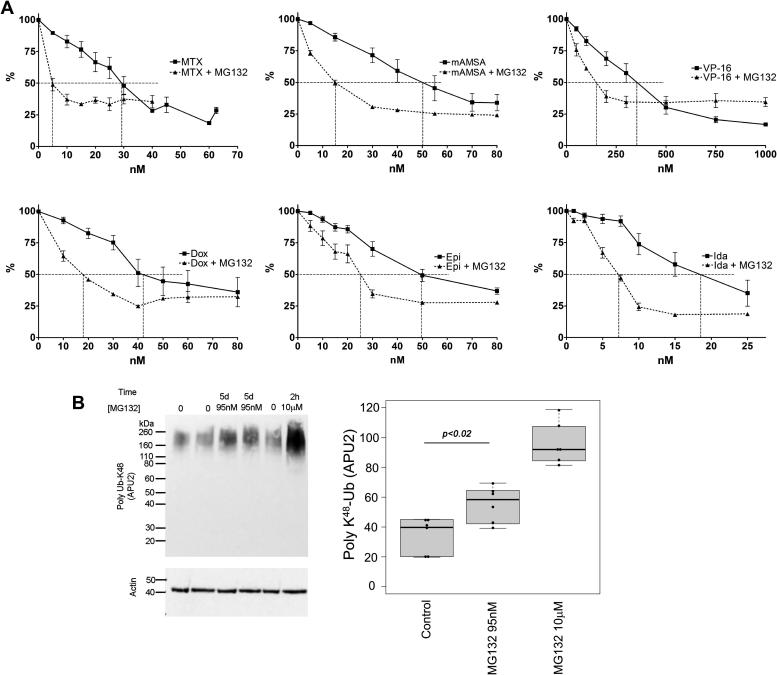
Potentiation of TOP2 poisons by MG132 in K562 cells. (A) Cells were treated with increasing concentrations of TOP2 poison alone or in combination with 95 nM MG132 for 5 days followed by XTT staining. Dose–response curves were used to estimate the IC_50_ of TOP2 poison alone and in combination with MG132. Error bars represent the mean ± SEM of at least 3 separate experiments for both + and − MG132 conditions, values were normalised to the 0 TOP2 poison value (100%). (B) 95 nM MG132 exposure over 5 days results in accumulation of K_48_-linked polyubiquitin consistent with compromised proteasome function. K562 cells were treated with MG132 at 95 nM for 5 days or 10 μM for 2 h. Western blots prepared from whole cell extracts were probed for K_48_ linked polyubiquitin. Blots representing six replicate treatments were scanned, densitometry values were normalised to actin and are expressed as a percentage of the mean normalised value obtained for 10 μM MG132.

**Fig. 3:**
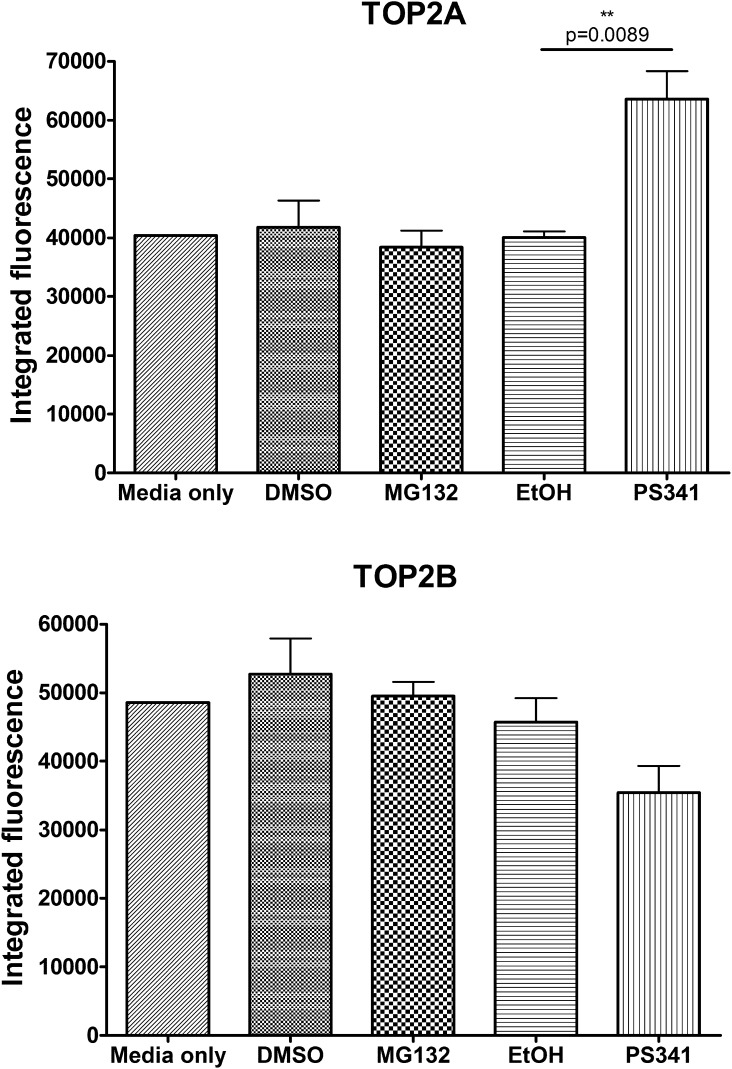
Effect of proteasome inhibitors on cellular TOP2 levels. K562 cells were seeded and incubated for 24 h, followed by treatment with 95 nM MG132 or 5.2 nM PS341 alone for 120 h as in the XTT assay. Cells were fixed onto poly lysine slides with 4% paraformaldehyde and TOP2A and TOP2B levels were quantified by immunofluorescence. Statistical comparisons were made between proteasome inhibitor alone and solvent control.

K562 cells were incubated with a combination of MG132 and one of six drugs that target DNA topoisomerase II. These included mitoxantrone, mAMSA and etoposide, (an anthracenedione, an acridine and an epipodophyllotoxin, respectively), and three anthracyclines; doxorubicin, its epimer epirubicin, and idarubicin. In order to investigate whether proteasome inhibition affected the growth inhibitory effect of these DNA topoisomerase II-targeting agents, K562 cells were treated with a range of concentrations of anti-topoisomerase II drug with or without MG132 and growth inhibition was measured by XTT staining. The concentration of MG132 employed was 95 nM, which on its own resulted in 20% growth inhibition under the conditions used (Fig. 1). The potentiation of TOP2 poisons by MG132 was considered significant if there was a statistically significant difference between the IC_50_ of TOP2 poison alone and the IC_50_ in the presence of MG132 (as determined by unpaired *t*-test). MG132 significantly potentiated growth inhibition by all six anti-topoisomerase II drugs (Fig.2A). Potentiation factors (Pf_50_) were calculated using the IC_50_ value of anti-topoisomerase II drug alone over the IC_50_ value of anti-topoisomerase II drug in combination with MG132, and are shown in Table 1. The greatest potentiation was observed with mitoxantrone and MG132, with a Pf_50_ of 4.58 (*p* ⩽ 0.0001). mAMSA showed the second strongest potentiation with a Pf_50_ of 2.68 (*p* = 0.0005). Similar levels of potentiation were observed between the three anthracyclines (Pf_50_ = 1.93, 1.93 and 1.92 for doxorubicin, epirubicin and idarubicin, respectively). The smallest potentiation was observed for etoposide with a Pf_50_ of 1.65, but this was also statistically significant (*p* = 0.0261). These data indicate that inhibiting the proteasomal protease with MG132 can potentiate four classes of drugs that target DNA topoisomerase II. To confirm that this dose of MG132 affects proteasome function, cells were incubated with 95 nM MG132 for 5 days, and accumulation of K48-linked polyubiquitin in the cells was determined by Western blotting with antibody APU2 [48] (Fig.2B). For comparison, cells were also incubated with 10 μM MG132 for 2 h. Western blotting with APU2 antibody resulted in a faint high molecular weight smear in untreated cells and a much more intense signal in cells exposed to 10 μM MG132, consistent with proteasomal inhibition and the resulting accumulation of polyubiquitinated proteins. Notably, in cells exposed to 95 nM MG132 for 5 days the APU2 signal was also significantly more intense than in control cells, indicating compromised proteasomal function at this dose of inhibitor. TOP2A and TOP2B protein levels were also measured in K562 cells following a 5 day incubation with 95 nM MG132 by quantitative immunofluorescence with rabbit anti-TOP2A (4566) and mouse anti-TOP2B (MAB6438). MG132 did not induce a change in TOP2A or TOP2B levels under these conditions, suggesting the observed potentiation of anti-topoisomerase II drugs by MG132 is not simply due to an increase in levels of drug target (Fig. 3).

### 3.2 Potentiation of anti-DNA topoisomerase drugs by the proteasome inhibitor PS341 in K562 cells

**Fig. 4:**
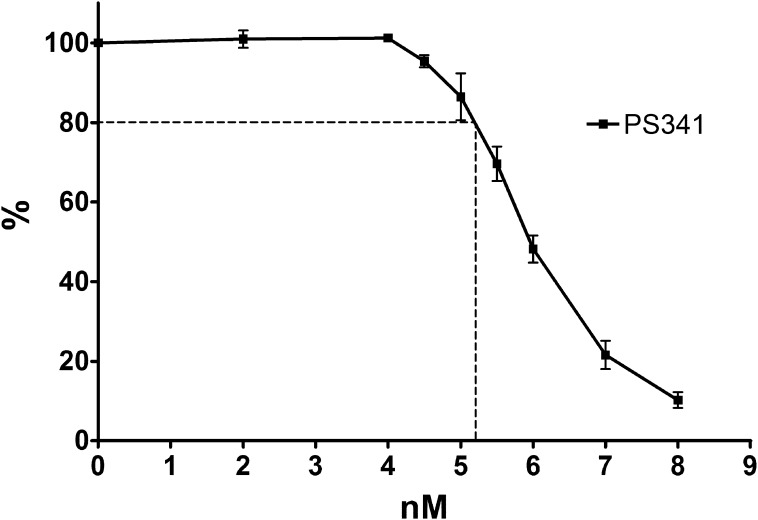
PS341 dose response in K562 cells. The IC_20_ of PS341 in K562 cells was determined by growth inhibition assays. Cells were treated with increasing concentrations of PS341 and stained after 5 days of incubation. Error bars represent the mean ± SEM of 3 separate experiments.

**Fig. 5:**
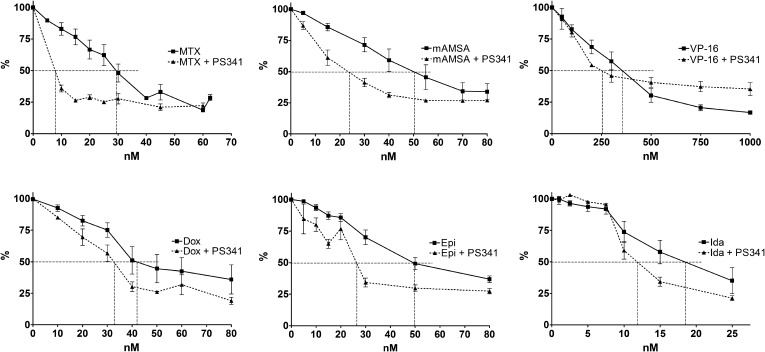
Potentiation of TOP2 poisons by PS341 in K562 cells. Cells were treated with increasing concentrations of TOP2 poison alone or in combination with 5.2 nM PS341 for 5 days followed by XTT staining. Dose–response curves (where % is inhibition of growth in relation to controls without TOP2 drug) were used to estimate the IC_50_ of TOP2 poison alone and in combination with PS341. Error bars represent the mean ± SEM of at least 3 separate experiments.

The proteasome inhibitor PS341 (bortezomib, Velcade) is used successfully in the clinic for multiple myeloma and is being trialled for other haematological malignancies [49]. To determine the effect of PS341 on the growth inhibitory effects of anti-DNA topoisomerase drugs, K562 cells were incubated with one of the above drugs alone or in combination with PS341. The IC_50_ values were determined as before and used to generate the Pf_50_ values, shown in Table 2. A fixed dose of 5.2 nM PS341 was used which on its own results in 20% growth inhibition (Fig. 4). PS341 significantly reduced the IC_50_ of mitoxantrone, epirubicin and mAMSA (*p* = 0.002, 0.0056 and 0.0259, respectively), but not etoposide, idarubicin or doxorubicin (*p* = 0.7690, 0.0890 and 0.1826) (Fig. 5). Mitoxantrone was potentiated most by PS341 with a Pf_50_ of 2.95. Notably, the level of potentiation with PS341 was lower than with MG132. Although both inhibitors primarily target the β5 subunit of the proteasome [50], MG132 is less selective than PS341 due to off-target inhibition of lysosomal cysteine proteases and the calpains [51]. Therefore, some MG132-mediated potentiation could be due to inhibition of proteases other than the proteasome. Nonetheless, the robust potentiation observed with PS341 and mitoxantrone, epirubicin or mAMSA indicates that the proteasome is an important determinant of the sensitivity of cells to these three TOP2 poisons.

It has been suggested that PS341 reduces resistance to TOP2 poisons by increasing levels of drug target, improving drug efficacy [37]. To investigate whether the potentiating effect of PS341 is due to an upregulation of TOP2 levels, K562 cells were incubated with 5.2 nM PS341 for 5 days. TOP2 levels were then measured by quantitative immunofluorescence. PS341 induced a significant increase in TOP2A levels (*p* = 0.0089) (Fig. 3). This is in contrast to MG132 which did not affect TOP2 levels. Thus, PS341 could potentiate TOP2 poison activity, at least partly by increasing the cellular level of TOP2A.

### 3.3 Role of TOP2A and TOP2B isoforms in the potentiation of anti-topoisomerase drugs by MG132

**Fig. 6:**
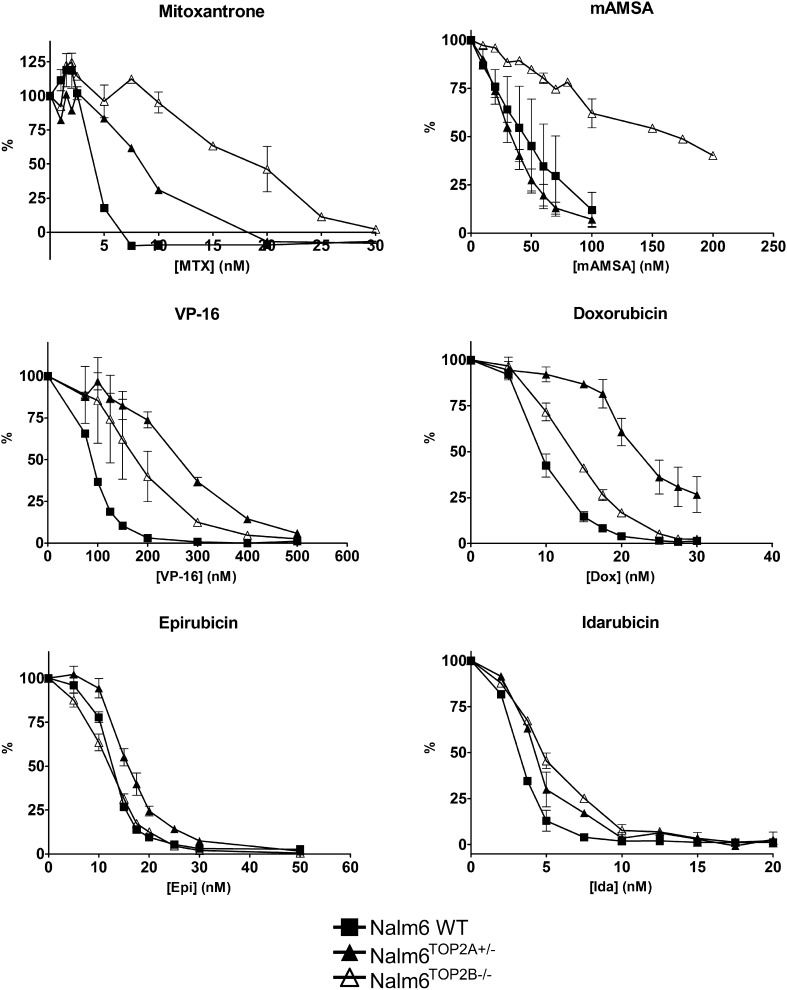
Growth inhibition of Nalm-6 WT, Nalm-6^TOP2A^^+/−^ and Nalm-6^TOP2B^^−/−^ cells by topoisomerase II poisons. Cells were treated with increasing concentrations of TOP2 poison and dose–response curves (where % is inhibition of growth in relation to untreated controls) were plotted. Error bars represent the mean ± SEM of at least 3 separate experiments.

To examine the role of TOP2A and TOP2B in the potentiation of anti-topoisomerase II drugs by MG132, growth inhibition assays were performed using the human pre-B cell line Nalm-6 and the *TOP2A^+/−^* and *TOP2B^−/−^* derivatives of Nalm-6 [38]. Compared to WT, Nalm-6 TOP2A expression is reduced to approximately 50% in Nalm-6^TOP2A^^+/−^ cells and TOP2B is absent in Nalm-6^TOP2B^^−/−^ cells. Notably, Nalm-6^TOP2B^^−/−^ cells were the most resistant to mitoxantrone and mAMSA compared to wild-type cells whilst Nalm-6^TOP2A^^+/−^ cells were most resistant to etoposide, doxorubicin and epirubicin. Nalm-6^TOP2A^^+/−^ and Nalm-6^TOP2B^^−/−^ cells were equally resistant to idarubicin (Fig. 6). These observations are consistent with those reported by Toyoda et al. [38] and Errington et al. [52] and support a relatively large role for TOP2B in the cytotoxic activity of mAMSA and mitoxantrone, and conversely a larger contribution of TOP2A in the cytotoxicity of etoposide and doxorubicin (Fig. 6).

**Fig. 7:**
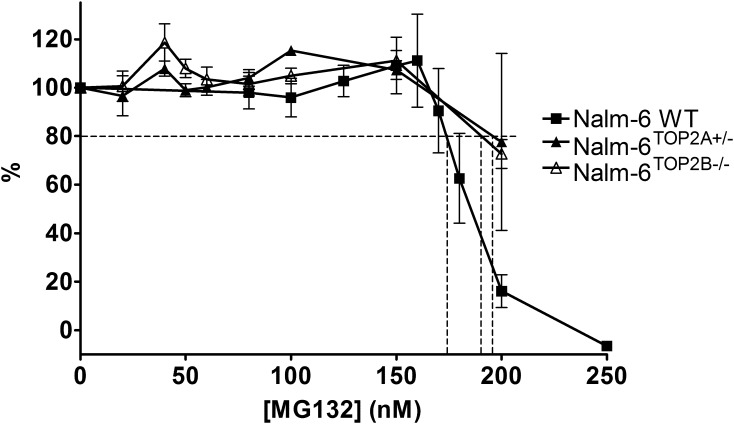
MG132 dose response in Nalm-6 WT, Nalm-6^TOP2A^^+/−^ and Nalm-6^TOP2B^^−/−^ cells. The IC_20_ of MG132 in Nalm-6 WT, Nalm-6^TOP2A^^+/−^ and Nalm-6^TOP2B^^−/−^ cells was determined by XTT assay. Cells were treated with increasing concentrations of MG132 and stained after 5 days of incubation. Error bars represent the mean ± SEM of at least 3 separate experiments.

**Fig. 8:**
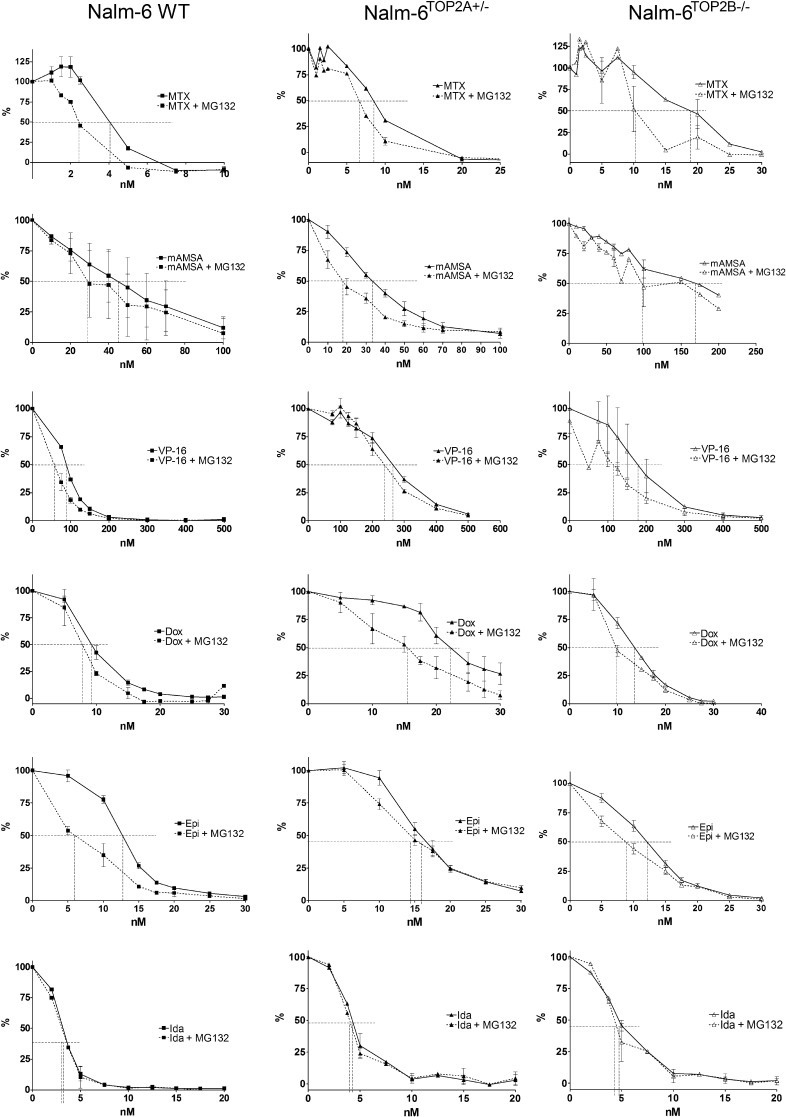
Potentiation of TOP2 poisons by MG132 in Nalm-6 WT, Nalm-6^TOP2A^^+/−^ and Nalm-6^TOP2B^^−/−^ cells. Cells were treated with increasing concentrations of TOP2 poison alone or in combination with 190 nM MG132 for 5 days followed by XTT staining. Dose–response curves (where % is inhibition of growth in relation to untreated controls) were used to estimate the IC_50_ of TOP2 poison alone and in combination with MG132. Error bars represent the mean ± SEM of at least 3 separate experiments.

For potentiation experiments in each of the Nalm-6 variant lines, 190 nM MG132 was used. This is the IC_20_ determined for MG132 in Nalm-6^TOP2B^^−/−^ cells, which did not differ significantly between the Nalm-6^TOP2B^^−/−^ cell line and that of Nalm-6^WT^ cells or between Nalm-6^TOP2B^^−/−^ and Nalm-6^TOP2A^^+/−^ cells (Fig. 7). Co-incubation with MG132 significantly reduced the IC_50_ of mitoxantrone, epirubicin and etoposide in wild type cells (*p* = 0.0173, 0.0237 and 0.0414, respectively; Fig. 8). As in K562 cells, this potentiation was greatest in combination with mitoxantrone (Pf_50_ = 1.94) (Table 3A, Table 3B). Interestingly, the potentiation of mitoxantrone was reduced (but remained significant) in Nalm-6^TOP2A^^+/−^ cells compared to wild type cells, suggesting TOP2A is involved in (but is not essential for) the potentiation of MTX with MG132. Unlike in wild type cells, there was no potentiation of etoposide or epirubicin in Nalm-6^TOP2A^^+/−^ cells, suggesting TOP2A is required for MG132-mediated potentiation of these two drugs.

There was also no potentiation of etoposide in Nalm-6^TOP2B^^−/−^ cells (*p* = 0.2560), suggesting a role for TOP2B in the potentiation of etoposide by MG132, whilst potentiation of epirubicin remained significant in Nalm-6^TOP2B^^−/−^ cells. In contrast, potentiation of mitoxantrone still occurred in both Nalm-6^TOP2A^^+/−^ cells and Nalm-6^TOP2B^^−/−^ cells. These results indicate that both isoforms are involved in the potentiation of anti-topoisomerase drugs by MG132, but the importance of each isoform differs between drugs. This may be due to the differential levels of TOP2 complexes formed by different anti-topoisomerase drugs [42].

### 3.4 Proteasomal inhibition affects TOP2-DNA complex reversal

**Fig. 9:**
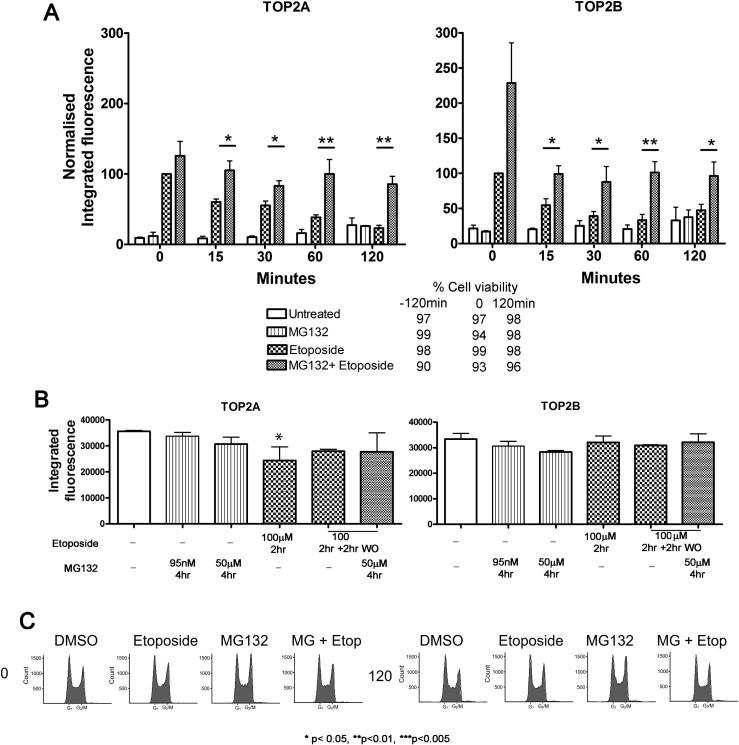
MG132 inhibits the reversal of etoposide-induced TOP2A- and TOP2B-DNA complexes. (A) K562 cells were incubated with solvent, etoposide (100 μM), MG132 (50 μM) or were co-incubated with 50 μM MG132 and 100 μM etoposide for 2 h. After 2 h etoposide was removed, but MG132 was maintained in cell incubations that initially contained it. Levels of TOP2A and TOP2B DNA complexes at 0, 15, 30, 60 and 120 min after etoposide removal (wash-out) were determined using the TARDIS assay. Statistical comparisons were made between the levels of TOP2-DNA complexes in the presence or absence of MG132 by unpaired *t*-test. Figures are given below for cell viability (trypan blue exclusion) under the cell treatment conditions used: “−120 min” refers to the time at which drugs were first added to cells, “0” refers to the time at which etoposide wash-out was performed and “120 min” refers to 2 h post drug wash-out. (B) The treatments employed in (A) do not significantly affect cellular TOP2A or TOP2B levels. Cells were treated as indicated and fixed with paraformaldehyde on poly lysine-coated slides. Total TOP2A and TOP2B was quantified by immunofluorescence. 100 μM etoposide incubation did result in a small decrease in total cellular TOP2A. (C) Cell cycle distribution at time of etoposide wash-out (“0”) and 2 h after etoposide washout (“120”).

**Fig. 10:**
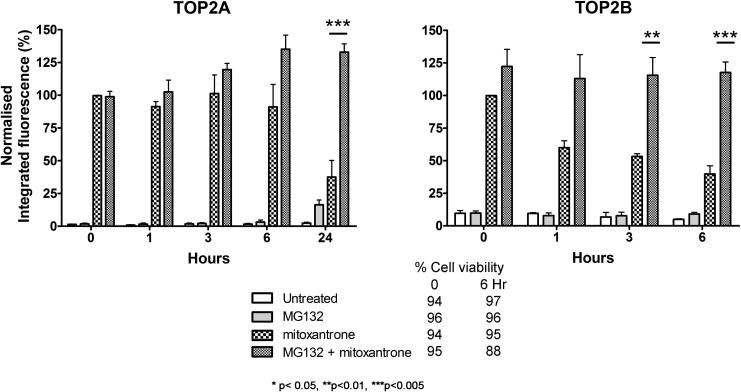
MG132 inhibits the reversal of mitoxantrone-induced TOP2A- and TOP2B-DNA complexes. K562 cells were incubated with solvent, mitoxantrone (5 μM), MG132 (50 μM) or were co-incubated with 50 μM MG132 and 5 μM mitoxantrone for 2 h. After 2 h mitoxantrone was removed, but MG132 was maintained in cell incubations that initially contained it. Levels of TOP2A and TOP2B DNA complexes at 0, 1, 3, 6 h after mitoxantrone removal (wash-out) were determined using the TARDIS assay and an additional time point of 24 h for TOP2A. Statistical comparisons were made between the levels of TOP2-DNA complexes in the presence or absence of MG132 by unpaired *t*-test. Figures are given below for cell viability (trypan blue exclusion) under the cell treatment conditions used: “−120 min” refers to the time at which drugs were first added to cells, “0” refers to the time at which mitoxantrone wash-out was performed and “1, 3, 6 h” refers to time post drug wash-out.

Inhibition of the proteasome is known to reduce resistance to chemotherapeutic agents by various mechanisms including inhibition of NFκB activation and cell cycle arrest [53]. In addition the proteasome plays a role in the processing of TOP2 protein-DNA complexes in response to TOP2 poisons [8], [9], [28], [32]. We measured the rate of reversal of etoposide-induced TOP2-DNA complexes in K562 cells in the presence or absence of the proteasome inhibitor MG312 (Fig. 9) and the rate of removal of mitoxantrone-induced TOP2-DNA complexes in K562 cells in the presence or absence of MG132 (Fig. 10). This was achieved using the TARDIS method [42], [43], [44] where covalent TOP2-DNA complexes are specifically measured by quantitative immunofluorescence of SDS-salt extracted agarose embedded cells (see Section 2). K562 cells were incubated with either etoposide (100 μM) or MG132 (50 μM) and etoposide (100 μM) concurrently for 2 h. Drug was then washed out and cells were incubated in fresh medium or fresh medium containing MG132 to maintain proteasomal inhibition. TOP2 complexes remained at background levels in cells incubated with MG132 alone (Fig.9A). When cells were incubated with etoposide, in the absence of MG132, the initially high TOP2A and TOP2B complex levels fell to background within 2 h of drug removal, as we have previously reported [21]. In the presence of MG132, although TOP2A fluorescence values initially dropped after etoposide removal, complex levels did not return to control levels during the time of the experiment, and this was statistically significant at all time points. Similar results were observed for TOP2B (Fig.9A). After etoposide washout the persistence of TOP2B complexes in the presence of MG132 was significant at all time points measured. During the time course of the experiment cell viability measured by trypan blue exclusion remained ⩾90% (Fig.9A). Fig.9B shows TOP2A and TOP2B protein levels determined by immunofluorescence for the time points used in the TARDIS experiment. There was a significant reduction in TOP2A protein levels after a 2 h exposure to etoposide, but this was no longer seen after 2 h in drug free media. Fig.9C shows FACS analysis of cell cycle distribution of the cells at 0 (2 h with etoposide or MG132 or etoposide and MG132) and 120 min (2 h after removal of etoposide). MG132 alone slightly increased the G2 fraction of cells at both time points, but cell cycle distribution was not significantly altered for other treatments during the time course of the experiment.

TOP2-DNA complexes stabilised by mitoxantrone have a longer half-life than those stabilised by etoposide, with TOP2A-DNA complexes being longer lived than TOP2B-DNA complexes [43]. Therefore, the rate of removal of mitoxantrone stabilised TOP2A- and TOP2B-DNA complexes was measured up to 24 h and 6 h after mitoxantrone removal, respectively (Fig. 10). Mitoxantrone was removed from the cell culture media after 2 h of drug treatment. In the absence of MG132 TOP2B complexes reduced over time: more than 50% of the complexes had reversed 6 h after removal of mitoxantrone, whilst in the presence of MG132 the TOP2B complex levels remained elevated. The proteasome inhibitor resulted in a significant persistence of TOP2B complexes at 3 and 6 h after mitoxantrone removal.

In contrast, even in the absence of MG132 the TOP2A complexes were not reduced at 1, 3 or 6 h. Mitoxantrone-stabilised TOP2A complexes took 24 h to reduce to less than 50% of the level seen after 2 h exposure to mitoxantrone. In the presence of MG132, the TOP2A complex levels remained elevated even at 24 h. This was statistically significant at 24 h compared to mitoxantrone alone (*p* < 0.005).

Thus, MG132 inhibits removal of drug stabilised TOP2-DNA complexes, confirming the role of the proteasome in the processing of TOP2 cleavable complexes. The proteasome may facilitate the removal of TOP2 from the DNA protein complex directly through degradation of TOP2 (as suggested by previous work), or indirectly by triggering repair pathways.

## 4 Discussion

In the current study, we have shown that proteasome inhibition by MG132 potentiates the growth inhibitory effects of six anti-topoisomerase II drugs (mitoxantrone, mAMSA, doxorubicin, epirubicin, idarubicin and etoposide) in K562 cells which are p53 null, and two or three anti-topoisomerase II drugs in Nalm-6 cell lines which are p53 wild type. In Nalm-6^WT^ cells MG132 potentiated the effect of mitoxantrone, etoposide and epirubicin; in Nalm-6^TOP2B^^−/−^ cells MG132 potentiated mitoxantrone, epirubicin and doxorubicin, whilst in Nalm-6^TOP2A^^+/−^ cells MG132 only potentiated mitoxantrone and mAMSA. Differences in the potentiation by MG132 with different anti-topoisomerase II drugs were observed. Potentiation of mitoxantrone with MG132 was seen in all 4 cell lines. This was greatest in K562 and mitoxantrone was the only agent potentiated in all three Nalm-6 cell lines. Both isoforms of TOP2 are targeted by mitoxantrone and it can stabilise protein-DNA complexes with either isoform [52]. Both isoforms seem to be required for MG132-mediated potentiation by etoposide as the differences in IC_50_ are not significant in Nalm-6^TOP2A^^+/−^ or Nalm-6^TOP2B^^−/−^ cells. Both TOP2A and TOP2B covalent complexes are trapped in response to etoposide [21]. Ours and other data suggest that TOP2A may be most important for etoposide-mediated cytotoxicity [38], [52] and growth inhibition (Fig. 7), as Nalm-6^TOP2^^+/−^ cells are the most resistant to etoposide. Nonetheless, Nalm-6^TOP2B^^−/−^ cells are also more resistant to etoposide than wild type cells, demonstrating TOP2B also plays a role in etoposide-mediated cytotoxicity.

MG132 inhibited the clearance of TOP2 covalent complexes stabilised by etoposide or mitoxantrone (Fig. 9, Fig. 10, respectively), increasing the levels of TOP2A and TOP2B covalent complexes. In the absence of MG132 the half-lives of TOP2A and TOP2B covalent complexes in response to mitoxantrone are 10 and 6 h respectively [52], compared to 30 min and 15 min respectively with etoposide [43]. With etoposide, MG132 increased the half-lives of TOP2A and TOP2B to >120 min, and with mitoxantrone increased the half-life of each isoform to >24 h and >6 h respectively. Thus, proteasome inhibition may potentiate TOP2 poisons by prolonging the half-life of TOP2-DNA complexes and preventing their repair. In support of this, it has been suggested that proteasome inhibition prevents the liberation of the TOP2-mediated DSB [8], which is required for the NHEJ repair of etoposide-induced DSBs [7]. Our data further support the role of the proteasome in the processing of TOP2-DNA complexes, which has been shown for both isoforms [8], [9], [28] and is consistent with Sunter et al. [54], who show that levels of TOP2-DNA complexes are increased by MG132 when compared to etoposide alone.

We also investigated the clinically used proteasome inhibitor PS341 (bortezomib, Velcade), the first proteasome inhibitor to be FDA-approved for clinical use in the treatment of relapsed or refractory multiple myeloma and mantle cell lymphoma, which also shows potential in clinical trials for use in combination with various chemotherapeutic agents [49]. We found PS341 potentiated the growth inhibition of three TOP2 poisons in K562 cells; mitoxantrone, mAMSA and epirubicin. We found that incubation with PS341 for 120 h significantly increased the level of TOP2A, supporting the suggestion that PS341 increases the sensitivity of cells to anti-topoisomerase II drugs by increasing levels of TOP2 in the cell, thereby increasing levels of drug target and drug efficacy [37]. Consistently, PS341 reduces resistance to anti-topoisomerase II drugs where TOP2 levels are reduced [36].

Notably, MG132 potentiated all six TOP2 poisons tested whilst PS341 potentiated only three TOP2 poisons. We cannot exclude that MG132 could potentiate anti-topoisomerase II drugs by other mechanisms. Proteasome inhibition perturbs the ubiquitination of substrates, leading to the accumulation of ubiquitin conjugates and the depletion of free ubiquitin [55]. Ubiquitination is important in the cellular response to DNA damage, co-ordinating the recruitment of DNA repair proteins. This is mediated largely by the E3 ubiquitin ligases RNF8 and RNF168 [56], [57], [58]. For example, the generation of K63-linked ubiquitin chains by RNF168 leads to the direct recruitment of BRCA1 via the ubiquitin-interacting motif (UIM) of Rap80 [59]. RNF168 also mediates the monoubiquitination of histone H2A at lysine 15 which is required for the recruitment of 53BP1 [60]. Importantly, MG132 was shown to inhibit BRCA1 and 53BP1 foci formation, as well as other key DNA repair proteins such as Rad51, in response to IR and cisplatin [61]. Therefore, MG132 and PS341 may potentiate the growth inhibitory effects of anti-topoisomerase II drugs by reducing repair of the double strand break once TOP2-DNA complexes are processed, as well as reducing the processing itself.

We have shown that inhibition of proteasomal activity potentiates the growth inhibitor effect of various TOP2 poisons in a myeloid and a lymphoblastic leukaemia cell line model. This potentiation is likely to involve increased accumulation and/or decreased clearing of TOP2 DNA complexes, although other mechanisms may also be involved. These findings support the notion that targeting the ubiquitin proteasome system in combination with existing therapies may be a productive approach. Furthermore, this may allow lower doses of anti-topoisomerase II drug to be used, which could potentially reduce unwanted genotoxic side effects, such as the occurrence of secondary leukaemias.

## Funding

This study was supported by BLOODWISE Research Specialist Program Grant No. 12031 and by a BLOODWISE Gordon Piller Studentship, 13063.

## Figures and Tables

**Table 1:** Pf_50_ values of TOP2 poisons in combination with the proteasome inhibitor MG132 in K562 cells. Cells were treated with increasing concentrations of TOP2 poison alone or in combination with 95 nM MG132 for 5 days and stained with XTT reagent. Pf_50_ values were calculated using the IC_50_ of TOP2 poison alone and the IC_50_ of TOP2 poison in combination with MG132. Pf_50_ values represent the mean of individual Pf_50_s from at least 3 separate experiments. *p* values are a comparison between IC_50_ of TOP2 poison alone versus the IC_50_ of TOP2 poison in combination with MG132.

	Mean Pf_50_	SEM	*p* value
Mitoxantrone	4.58	0.54	<0.0001
mAMSA	2.68	0.30	0.0005
Etoposide	1.65	0.25	0.0261
Doxorubicin	1.93	0.31	0.0155
Epirubicin	1.93	0.24	0.0018
Idarubicin	1.92	0.09	0.0018

**Table 2:** Pf_50_ values of TOP2 poisons in combination with the proteasome inhibitor PS341 in K562 cells. Cells were treated with increasing concentrations of TOP2 poison alone or in combination with 5.2 nM PS341 for 5 days and stained with XTT reagent. Pf_50_ values were calculated using the IC_50_ of TOP2 poison alone and the IC_50_ of TOP2 poison in combination with PS341. Pf_50_ values represent the mean of individual Pf_50_s from 3 separate experiments. *p* values are a comparison between IC_50_ of TOP2 poison alone versus the IC_50_ of TOP2 poison in combination with PS341.

	Mean Pf_50_	SEM	*p* value
Mitoxantrone	2.95	0.11	0.002
mAMSA	1.70	0.35	0.0259
Etoposide	1.24	0.32	0.7690
Doxorubicin	1.44	0.23	0.1826
Epirubicin	1.63	0.05	0.0056
Idarubicin	1.37	0.02	0.0890

**Table 3A:** TOP2 poison IC_50_ values alone and in combination with MG132 in Nalm-6 cell lines. Cells were treated with increasing concentrations of TOP2 poison alone or in combination with 190 nM MG132 for 5 days and stained with XTT reagent. IC_50_ values represent the mean IC_50_ from at least 3 separate experiments.

	Nalm-6 WT		Nalm-6^TOPA2+/−^		Nalm-6^TOP2B−/−^	
	IC_50_ alone (nM)	IC_50_ with MG132 (nM)	IC_50_ alone (nM)	IC_50_ with MG132 (nM)	IC_50_ alone (nM)	IC_50_ with MG132 (nM)
Mitoxantrone	4.23	2.33	8.67	6.57	17.43	6.10
mAMSA	49.50	39.17	33.92	19.33	152.50	113.08
Etoposide	85.67	59.33	264.33	235.67	156.80	126.60
Doxorubicin	9.43	7.17	23.30	14.00	13.53	9.60
Epirubicin	12.80	7.40	16.10	14.37	12.07	9.03
Idarubicin	3.03	2.87	3.92	3.46	4.77	3.95

**Table 3B:** Pf_50_ values of TOP2 poisons in combination with MG132 in Nalm-6 WT, Nalm-6^TOP2A^^+/−^ and Nalm-6^TOP2B^^−/−^ cells. *p* values are a comparison between the IC_50_ of TOP2 poison alone versus the IC_50_ of TOP2 poison in combination with MG132. Pf_50_ values represent the mean of individual Pf_50_ values from at least 3 separate experiments (MTX *n* = 3, mAMSA *n* = 6, etoposide *n* = 3, doxorubicin *n* = 3, epirubicin *n* = 3, idarubicin *n* = 6).

	Nalm-6 WT			Nalm-6^TOP2A+/−^			Nalm-6^TOP2B−/−^		
	Mean Pf_50_	SEM	*p* value	Mean Pf_50_	SEM	*p* value	Mean Pf_50_	SEM	*p* value
Mitoxantrone	1.94	0.33	0.0173	1.32	0.05	0.0021	2.93	0.35	0.0002
mAMSA	1.29	0.06	0.4554	2.02	0.36	0.0017	1.25	0.12	0.1710
Etoposide	1.46	0.06	0.0414	1.12	0.04	0.1177	1.24	0.18	0.2560
Doxorubicin	1.42	0.36	0.1472	1.73	0.26	0.0560	1.42	0.09	0.0135
Epirubicin	1.88	0.37	0.0237	1.12	0.05	0.1770	1.35	0.08	0.0318
Idarubicin	1.06	0.03	0.3653	1.14	0.07	0.3206	1.25	0.13	0.1717
